# *HOS2* and *HDA1* Encode Histone Deacetylases with Opposing Roles in *Candida albicans* Morphogenesis

**DOI:** 10.1371/journal.pone.0012171

**Published:** 2010-08-13

**Authors:** Lucia F. Zacchi, Wade L. Schulz, Dana A. Davis

**Affiliations:** Department of Microbiology, University of Minnesota, Minneapolis, Minnesota, United States of America; Duke University, United States of America

## Abstract

Epigenetic mechanisms regulate the expression of virulence traits in diverse pathogens, including protozoan and fungi. In the human fungal pathogen *Candida albicans*, virulence traits such as antifungal resistance, white-opaque switching, and adhesion to lung cells are regulated by histone deacetylases (HDACs). However, the role of HDACs in the regulation of the yeast-hyphal morphogenetic transitions, a critical virulence attribute of *C. albicans*, remains poorly explored. In this study, we wished to determine the relevance of other HDACs on *C. albicans* morphogenesis. We generated mutants in the HDACs *HOS1*, *HOS2*, *RPD31*, and *HDA1* and determined their ability to filament in response to different environmental stimuli. We found that while *HOS1* and *RPD31* have no or a more limited role in morphogenesis, the HDACs *HOS2* and *HDA1* have opposite roles in the regulation of hyphal formation. Our results demonstrate an important role for HDACs on the regulation of yeast-hyphal transitions in the human pathogen *C. albicans*.

## Introduction

*Candida albicans* is the most common fungal pathogen of humans and is the fourth most common cause of nosocomial bloodstream infections [1]. *C. albicans* pathogenesis depends on its ability to transition between the yeast, pseudophyphal, and hyphal cellular morphologies [2], and these transitions are triggered by diverse environmental cues, including temperature, serum, pH, and starvation [3]. Both the yeast and hyphal morphologies are required for pathogenesis in animal models of infection [4]–[6], and are required for the formation of normal biofilms [7], [8], a structure that increases antifungal drug resistance and constitutes a source of inoculum for disseminated and recurrent infections [9]. The different cellular morphologies can also trigger immune tolerance or activation against *C. albicans* [10]–[12]. Therefore, the ability to switch between morphologies has pleiotropic effects on *C. albicans* interaction with the host and on its ability to cause infection.

As epigenetic regulators of gene expression, chromatin modifying enzymes regulate diverse aspects of *C. albicans* biology. For example, histone modifying enzymes are required for the regulation of virulence traits and for pathogenesis in *C. albicans* [13]–[23]. Since the yeast-hyphal switch is critical for pathogenesis, we investigated the role of histone deacetylases (HDACs) in the regulation of this virulence trait. Here, we screened mutants in *HOS1*, *HOS2*, *RPD31*, and *HDA1* for a role in *C. albicans* morphogenesis. We found that *HOS1* and *RPD31* have little to no role in morphogenesis, and that *HOS2* and *HDA1* encode proteins with opposing roles in morphogenesis: Hos2 functions as a repressor, while Hda1 functions as an inducer of filamentation.

## Results

Chromatin remodeling proteins effect diverse aspects of *C. albicans* biology. Several histone modifying enzymes in *C. albicans*, including the histone methyltransferase Set1 and the histone acetyl transferase complex NuA4, are required for the expression of virulence factors and for pathogenesis *in vivo* [16], [21]. The yeast-to-hyphal transition is one biological property of *C. albicans* required for pathogenesis, and it is governed at least in part by epigenetic processes [16], [22]. To further address the role of chromatin remodeling proteins and epigenetic regulation on pathogenesis, we investigated the role of HDACs in the yeast-to-hyphal transition.

**Figure 1:**
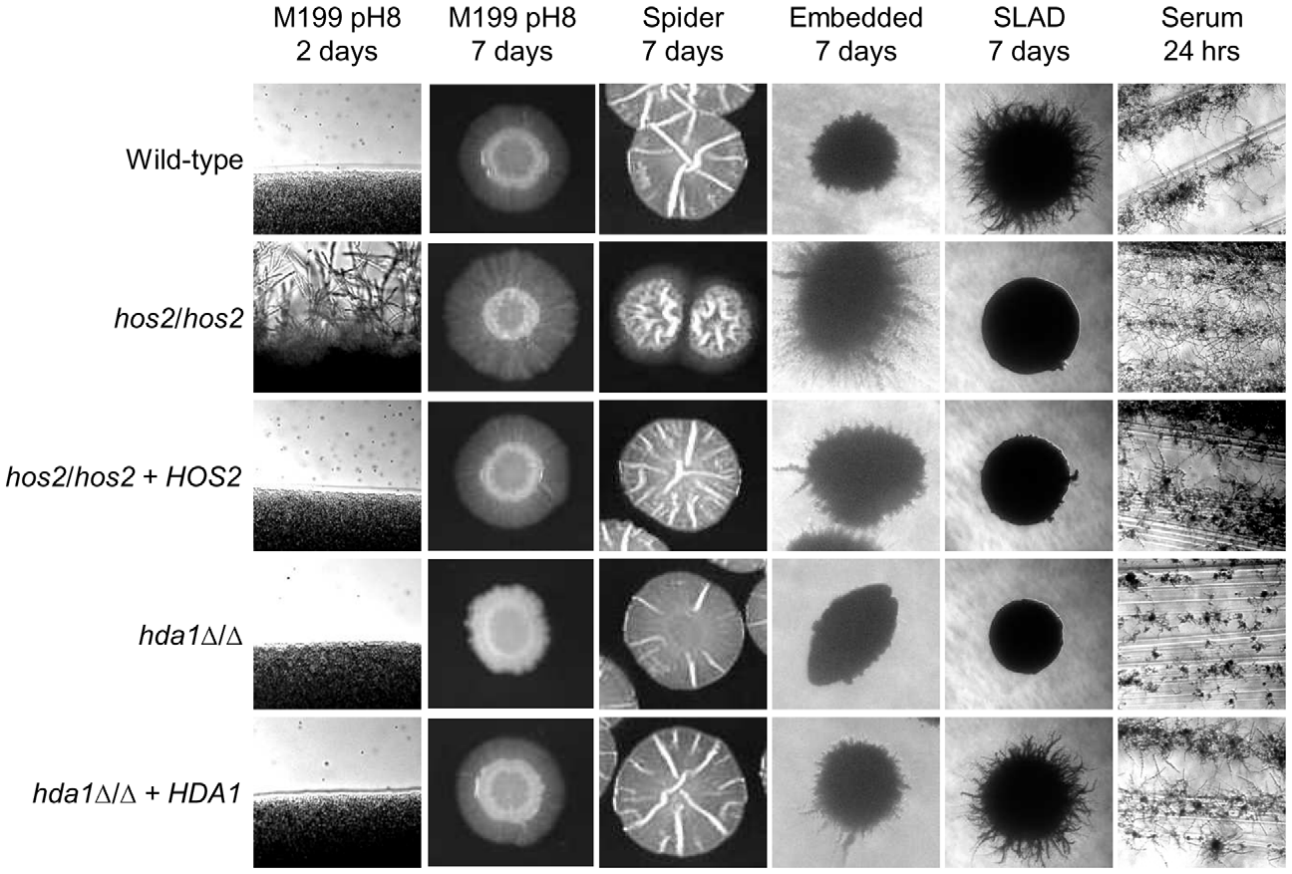
HDACs regulate filamentation on solid media. Overnight cultures of *C. albicans* wild-type (DAY185), *hos2/hos2* (DAY1252), *hos2/hos2 +HOS2* (DAY1250), *hda1Δ/Δ* (DAY1240), and *hda1Δ/Δ +HDA1* (DAY1241) strains grown in YPD at 30°C were: spotted onto M199 buffered to pH 8; serially diluted in PBS up to ∼100 CFU and plated on Spider and SLAD media; diluted 1∶1000 in 2 ml fresh YPD, incubated 4 hrs at 30°C and 8 µl were plated in embedded agar; streaked in synthetic complete medium supplemented with 4% bovine calf serum (BCS). All plates were incubated at 37°C, except for embedded agar which was incubated at 23°C. A minimum of three independent repetitions for each filamentation assay was performed.

**Figure 2:**
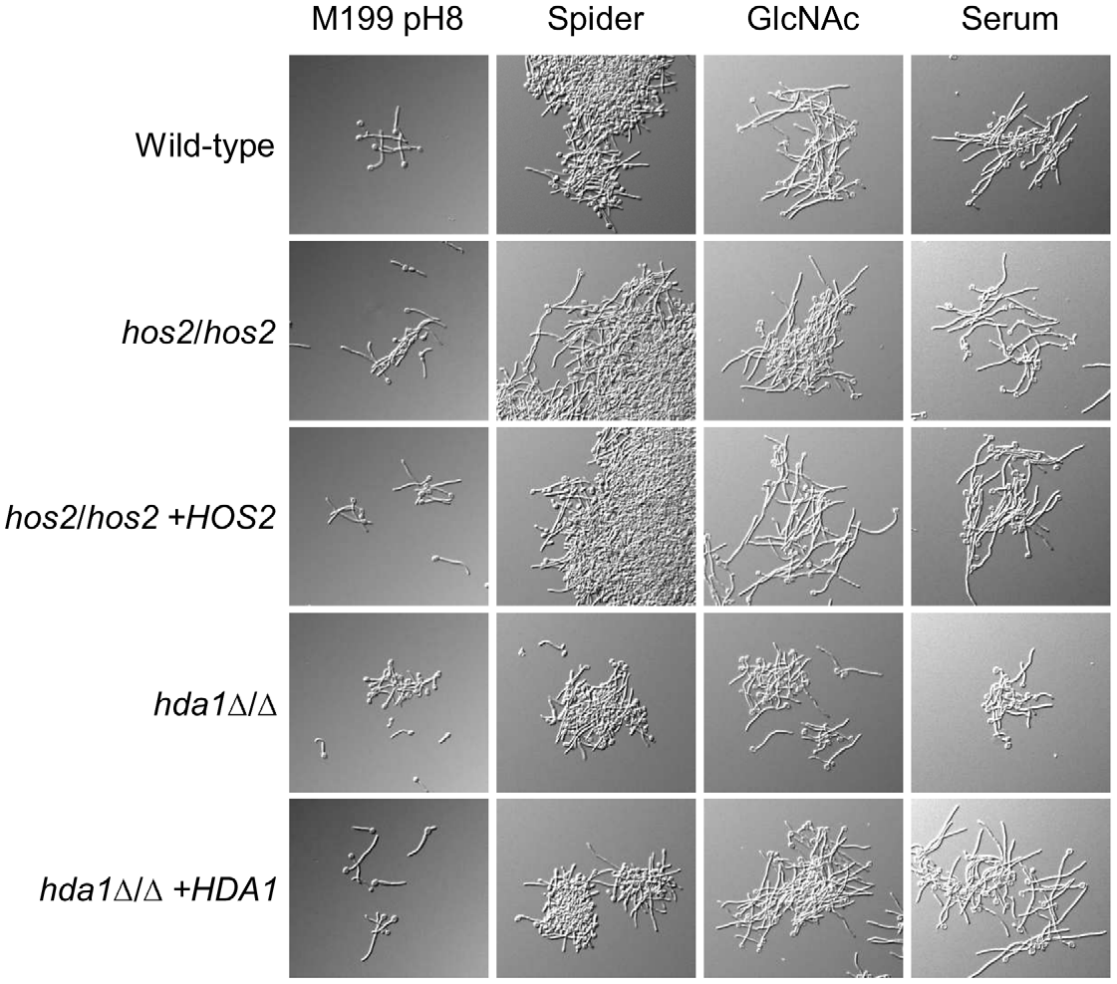
HDACs regulate filamentation in liquid media. Overnight YPD cultures of *C. albicans* wild-type (DAY185), *hos2/hos2* (DAY1252), *hos2/hos2 +HOS2* (DAY1250), *hda1Δ/Δ* (DAY1240), and *hda1Δ/Δ +HDA1* (DAY1241) strains were washed in PBS, diluted 1∶100 in M199 pH 8, Spider, YP+10% BCS, and YP+0.5% GlcNAc media and incubated 3 hrs at 37°C.

We identified Tn7::*UAU1* insertion clones located close to the START codon of *HOS1* (orf19.4411), *HOS2* (orf19.5377), and *RPD31* (orf19.6801) (Table 1). When available, two clones were used to disrupt the same gene to enhance the robustness of the approach. (Tn7::*UAU1* insertions were identified within additional HDACs, but these plasmids had complex or incomplete inserts (data not shown)). We generated *hos1/hos1, hos2/hos2,* and *rpd31/rpd31* mutants using the Tn7::*UAU1* insertional mutagenesis system [24]. The *hda1*Δ/Δ mutant was generated by sequential gene deletion using auxotrophic markers (Table 1). All mutants were tested for filamentation in solid and liquid media (Figures 1 and 2 and Table 2). Since *HOS1* and *RPD31* had little effect on filamentation (data not shown), we only describe the results for the *hos2/hos2* and *hda1*Δ/Δ mutants.

**Table 1:** List of mutants in histone deacetylases, the mutagenesis strategies, and corresponding TIGR CAG clones.

ORF19	Gene	Clone ID	Mutagenesis strategy	pDDB#	Strain
orf19.4411	*HOS1*	36246	Tn7 insertion clone CAGLH56	362	DAY1249
orf19.5377	*HOS2*	51640	Tn7 insertion clone CAGN203	363	DAY1242
orf19.5377	*HOS2*	17390	Tn7 insertion clone CAGFC21	357	DAY1243
orf19.2772	*HOS3*	65221	Tn7 insertion clone CAGR472	365	DAY1248
orf19.6801	*RPD31*	38517	Tn7 insertion clone CAGJX54	361	DAY1247
orf19.6801	*RPD31*	32377	Tn7 insertion clone CAGH755	358	DAY1246
orf19.2606	*HDA1*	-	Start-to-stop deletion		DAY694

**Table 2:** Germ tube formation delay of the *hda1*Δ/Δ mutant in M199 pH 8 and Spider media.

Strain		% germ tube ± SE	
		M199 pH 8	Spider
DAY185	Wild-type	62.5±2.4	71.2±4.3
DAY1252	*hos2/hos2*	69.8±2.8	71.0±4.2
DAY1250	*hos2/hos2* + *HOS2*	66.1±3.4	77.5±3.2
DAY1240	*hda1*Δ/Δ	27.3±2.8**	55.2±3.8*
DAY1241	*hda1*Δ/Δ + *HDA1*	67.2±2.2	72.7±2.5

Mean (% germ tubes) ± SE (Standard Error) of two independent experiments (n = 6). * p<0.03, ** p<0.003. Statistical analysis was performed using two tailed, paired T-Test.

Several different environmental conditions induce the hyphal morphology in *C. albicans*. Incubation at body temperature (37°C), alkaline pH, starvation, and serum are some of the signals that trigger hyphal morphogenesis in this fungus [3]. Further, incubation on solid surfaces, liquid media, or embedment in a matrix also impact *C. albicans* morphogenetic responses [25], [26]. Thus, we tested the ability of the HDACs mutants to filament in several different environmental conditions, including solid and liquid M199 pH 8, serum, and Spider media, solid SLAD medium, embedded agar, and liquid media supplemented with GlcNAc. The *hos2/hos2* mutants consistently showed enhanced filamentation compared to the wild-type strain on most solid media tested (Figure 1). On M199 pH 8, the *hos2/hos2* mutants filamented robustly, and showed a homogeneous peripheral halo of filamentation after 48 hrs of incubation, ∼24 hrs earlier than the wild-type strain (Figure 1 and data not shown). Similar results were observed on Spider medium, in embedded agar, and on serum (Figure 1). On SLAD, however, the *hos2/hos2* mutants showed either no filamentation or irregular filamentation around some colonies (Figure 1 and data not shown). Complementation of the *hos2/hos2* mutation restored filamentation to wild-type levels in all media except SLAD. Lack of complementation on SLAD medium may indicate haploinsufficiency of *HOS2*, as reported previously for other mutants grown on SLAD, such as *gap1*Δ*/*Δ and *gpr1Δ/Δ* [27], [28]. An independent *hos2*Δ/Δ start-to-stop deletion mutant also showed enhanced filamentation, corroborating the results of the insertional mutations (data not shown). Thus, Hos2 functions as an inhibitor of filamentation, except in conditions of nitrogen starvation (SLAD) in which Hos2 function is required for morphogenesis.

The *hda1*Δ/Δ mutant showed poor filamentation compared to the wild-type strain on most solid media tested (Figure 1). On M199 pH 8 and SLAD, the *hda1*Δ/Δ mutant did not filament. On Spider medium, the *hda1*Δ/Δ mutant showed a slight but reproducible smoother surface than the wild-type strain. In embedded agar, the *hda1*Δ/Δ mutant showed poor filamentation. On serum, the *hda1*Δ/Δ mutant showed a slight defect in hyphal formation. Complementation of the *hda1*Δ/Δ mutation restored filamentation to wild-type on M199 pH 8, Spider, embedded, and serum media, and partially rescued the defects on SLAD. Thus, Hda1 functions as an inducer of filamentation.

In liquid media, the *hos2/hos2* strain filamented similarly to wild-type in all media tested (Figure 2 and Table 2). The *hda1*Δ/Δ mutant also filamented in all media tested, but the filaments of the *hda1*Δ/Δ mutant appeared shorter than wild-type. Accordingly, we detected a delay in *hda1*Δ/Δ mutant germ tube formation in M199 pH 8 and Spider media compared to the wild-type, *hos2/hos2,* and *hda1*Δ*/*Δ*+HDA1* strains (Table 2). We noted that the results obtained in liquid media were more variable compared to solid media. Since changes in gene silencing occurs over several generations [29], [30], the rapid induction of filamentation in liquid medium may be more susceptible to variations than in solid media because of the differences in incubation time (<1 hr *vs* >24 hrs, respectively). This difference between liquid and solid medium filamentation may also be due to the fact that liquid filamentation is assessed at the single cell level while solid filamentation is assessed at the population (colony) level [29]. While the requirement for several generations in order for silencing to be altered may explain the disparate results for the *hda1Δ/Δ* mutant in solid *vs*. liquid media, it is also possible that Hda1 might be associated with regulators of filamentation that play a more prominent role in solid compared to liquid media. Differences in the function of regulators of hyphal formation in *C. albicans* when cells are incubated in solid, semi-solid, or liquid media have been previously described [25], [26], [31], [32]. Overall, our results demonstrate that the HDACs *HOS2* and *HDA1* have opposing roles in the regulation of hyphal formation in *C. albicans*.

## Discussion

Epigenetic mechanisms regulate virulence traits of diverse microbes, including *Trypanosoma brucei* and *Candida glabrata* [33], [34]. Epigenetic mechanisms also regulate aspects of *C. albicans* pathogenesis. Set1, a histone methyltransferase, the chromatin remodeling complex Swi/Snf, the histone acetyltransferase NuA4 complex, and the HDAC Sin3 regulate morphogenesis, adherence to epithelial cells, and/or are required for pathogenesis in animal models [16], [21], [35]. Furthermore, histone acetylation, regulated by the SAGA/ADA coactivator complex is required for the proper response to oxidative stress and antifungals [23]. White-opaque switching is regulated by transcriptional feedback loops and HDACs [13], [15], [19], [36]. HDACs function is also required for antifungal resistance and adhesion to human pneumocytes [14], [17], [18], [20]. Therefore, epigenetic mechanisms play an important role in the pathogenesis of *C. albicans*.

Here, we show that Hos2 and Hda1 regulate the yeast-to-hyphal transition in opposing ways. Previously, Hos2 and Hda1 were reported to have opposing effects on white-opaque switching [15], [19]. This suggests that Hos2 and Hda1 may inversely govern a common set of genes. Histone deacetylation is usually associated with transcriptional repression [37], [38]. However, HDACs are also required for gene expression, and it has been proposed that acetylation and deacetylation cycles are responsible for maintaining promoter activity [39]–[41]. HDACs can deacetylate histones globally (non-targeted deacetylation) or at specific promoters to which they are tethered in complex with specific transcription factor and other DNA binding proteins (targeted deacetylation) [42], [43]. Thus, one possible mechanisms of Hos2 and Hda1 function on filamentation in *C. albicans* is through the association with transcriptional regulators of hyphal formation, including the positive regulators Cph1, Cph2, Efg1, Tec1, Bcr1, Czf1, and/or Rim101, and the negative regulators Nrg1, Tup1, Rfg1, and/or Sfl1 [3], [25], [44]–[47]. For example, Hos2 and Hda1 have been associated with Tup1 and Efg1 function in *S. cerevisiae* and *C. albicans*, respectively. [48], [49], [50]. HDACs could also impact filamentation by affecting the expression of the regulators themselves [19], [22] or by deacetylating transcription factors and other non-histone proteins that have a direct or indirect role in morphogenesis [40], [43], [51]–[55]. Thus, Hos2 and Hda1 might impact hyphal formation through a diverse array of mechanisms.

Why is *HOS2* required for filamentation in SLAD but acts as an inhibitor of hyphal formation in all other conditions tested? In *C. albicans*, hyphal formation on SLAD is modulated by transcription factors, some of which function specifically during nitrogen starvation, such as Gln3. It is possible that Hos2 is required for the function of these specific transcription factors. Alternatively, loss of Hos2 may promote expression of genes that inhibit morphogenesis during nitrogen starvation. Thus, the *hos2Δ/Δ* effect on morphogenesis in *C. albicans* varies with the environmental conditions, a phenomenon that has also been observed for the histone deacetylase Set3 [50].

HDAC inhibitors have been proposed as antifungal adjuvants, due to their effect on preventing antifungal resistance *in vitro* [14], [17], [20]. However, no studies have shown the efficacy of HDAC inhibitors as antifungals *in vivo*. These types of experiments become even more critical in lieu of our and others findings that HDACs have differential effects on hyphal formation. Previous reports show conflicting *in vitro* results on the effect of different HDAC inhibitors on germ tube formation in liquid serum [14], [18]. However, inhibiting HDAC function could enhance filamentation in semi-solid surfaces (Figure 1) (such as mucosas), possibly leading to enhanced tissue invasion and biofilm formation, with the potential to cause more damage and increase antifungal resistance [56]. On the contrary, the use of specific HDAC inhibitors might enhance antifungal effectiveness by limiting hyphal development (e.g. against Hda1 (Figure 1)), or by limiting yeast development (e.g. against Hos2 (Figure 1) and [55]). The critical role of HDACs in *C. albicans* pathogenesis and survival to antifungal treatment underscores the necessity to study HDAC function in this organism. A combination of *in vitro* and *in vivo* studies that assess the role of HDACs in biofilm development, genomic instability, colonization, survival, and pathogenesis could determine the potential of HDAC inhibitors as antifungal drugs. Overall, our results contribute to demonstrate the importance of epigenetic regulators in governing virulence traits in *C. albicans*, and support the potential of HDAC inhibitors to prevent and/or treat candidal infections.

## Materials and Methods

### Strains and plasmids

All *C. albicans* strains used in this study derive from *C. albicans* strain BWP17 (Table 3). The *hos1*/*hos1*, *hos2/hos2*, and *rpd31/rpd31* strains were generated using the Tn7::*UAU1* insertional mutagenesis system [24] using clones obtained from TIGR. Mutagenesis and selection of Tn7::*UAU1* transformants was performed using primers in Table 4 as previously described [24]. The *hda1*Δ*/*Δ mutant DAY694 was constructed by sequentially deleting both *HDA1* alleles from the start to the stop codon from BWP17 strain, using *hda1::ARG4* and *hda1::URA3-dpl200* disruption cassettes PCR amplified with primers HDA1 5DR and HDA1 3DR (Table 4). The complemented and prototrophic strains (Table 3) were constructed by transformation with NruI digested plasmids pDDB503 for *HOS2* complementation, pDDB504 for *HDA1* complementation, and empty vector pDDB78.

**Table 3:** *C. albicans* strains.

Strain	Parent/Background	Genotype	Reference
DAY1 (BPW17)	SC5314	*ura3::λimm434/ura3::λimm434 his1::hisG/his1::hisG arg4::hisG/arg4::hisG*	[59]
DAY185	DAY286	*ura3::λimm434/ura3::λimm434 pHIS1::his1::hisG/his1::hisG ARG4::URA3::arg4::hisG/arg4::hisG*	[24]
DAY1242	DAY1	*ura3::λimm434/ura3::λimm434 his1::hisG/his1::hisG arg4::hisG/arg4::hisG hos2::Tn7::ARG4/hos2::Tn7::URA3*	This study
DAY1243	DAY1	*ura3::λimm434/ura3::λimm434 his1::hisG/his1::hisG arg4::hisG/arg4::hisG hos2::Tn7::ARG4/hos2::Tn7::URA3*	This study
DAY1246	DAY1	*ura3::λimm434/ura3::λimm434 his1::hisG/his1::hisG arg4::hisG/arg4::hisG rpd31::Tn7::ARG4/ rpd31::Tn7::URA3*	This study
DAY1247	DAY1	*ura3::λimm434/ura3::λimm434 his1::hisG/his1::hisG arg4::hisG/arg4::hisG rpd31::Tn7::ARG4/ rpd31::Tn7::URA3*	This study
DAY1249	DAY1	*ura3::λimm434/ura3::λimm434 his1::hisG/his1::hisG arg4::hisG/arg4::hisG hos1::Tn7::ARG4/hos1::Tn7::URA3*	This study
DAY694	DAY1	*ura3::λimm434/ura3::λimm434 his1::hisG/his1::hisG arg4::hisG/arg4::hisG hda1::ARG4/hda1::URA3-dpl200*	This study
DAY1250	DAY1242	*ura3::λimm434/ura3::λimm434 pHIS1::HOS2::his1::hisG/his1::hisG arg4::hisG/arg4::hisG hos2::Tn7::ARG4/hos2::Tn7::URA3*	This study
DAY1251	DAY1243	*ura3::λimm434/ura3::λimm434 pHIS1::HOS2::his1::hisG/his1::hisG arg4::hisG/arg4::hisG hos2::Tn7::ARG4/hos2::Tn7::URA3*	This study
DAY1252	DAY1242	*ura3::λimm434/ura3::λimm434 pHIS1::his1::hisG/his1::hisG arg4::hisG/arg4::hisG hos2::Tn7::ARG4/hos2::Tn7::URA3*	This study
DAY1253	DAY1243	*ura3::λimm434/ura3::λimm434 pHIS1::his1::hisG/his1::hisG arg4::hisG/arg4::hisG hos2::Tn7::ARG4/hos2::Tn7::URA3*	This study
DAY1241	DAY694	*ura3::λimm434/ura3::λimm434 pHIS1::HDA1::his1::hisG/his1::hisG arg4::hisG/arg4::hisG hda1::ARG4/hda1::URA3-dpl200*	This study
DAY1240	DAY694	*ura3::λimm434/ura3::λimm434 pHIS1::his1::hisG/his1::hisG arg4::hisG/arg4::hisG hda1::ARG4/hda1::URA3-dpl200*	This study
DAY1305	DAY1249	*ura3::λimm434/ura3::λimm434 pHIS1::his1::hisG/his1::hisG arg4::hisG/arg4::hisG hos1::Tn7::ARG4/hos1::Tn7::URA3*	This study
DAY1306	DAY1246	*ura3::λimm434/ura3::λimm434 pHIS1::his1::hisG/his1::hisG arg4::hisG/arg4::hisG rpd31::Tn7::ARG4/ rpd31::Tn7::URA3*	This study
DAY1307	DAY1247	*ura3::λimm434/ura3::λimm434 pHIS1::his1::hisG/his1::hisG arg4::hisG/arg4::hisG rpd31::Tn7::ARG4/ rpd31::Tn7::URA3*	This study
DAY414 (L40)	*S. cerevisiae*	*MATα his3*Δ*200 trp1-901 leu2-3,-112 ade2 LYS2::(lexAop)_4_-HIS3 URA3::(lexAop)_8_-lacZ GAL4*	[60]

**Table 4:** Primers used in this study.

Name	Sequence (5′to 3′)	Reference
HOS2 DDB78 comp 5′	acgacggccagtgaattgtaatacgactcactatagggcgccaatcacagaactcaaggc	This study
HOS2 DDB78 comp 3′	aagctcggaattaaccctcactaaagggaacaaaagctggctatcttgttaattgatggg	This study
HDA1 5′ comp	aagctcggaattaaccctcactaaagggaacaaaagctggtcatctgctctccattgacg	This study
HDA1 3′ comp	acgacggccagtgaattgtaatacgactcactatagggcggaatttaatgaacccgatgg	This study
HDA1 5-1 comp	atatatccatatccggctgg	This study
HDA1 3-1 comp	ttctggtatgcacgacggtg	This study
ARG4-detect	ggaattgatcaattatcttttgaac	This study
FC21 5detect	tttttacaatcgataactcc	This study
FC21 3detect	acgttggttgaaatttcgtg	This study
N203 5detect	ccaatatataaccataggag	This study
N203 3detect	ggcaatatctgacattcctg	This study
H755 5detect	gctgatattgggaattatgc	This study
H755 3detect	gcttcttcaacaccatcacc	This study
JX54 5detect	gccgccagattgaatcgtgg	This study
JX54 3detect	ccaccaactaccatcattgg	This study
R472 5detect	gcatttgaaacaacattgac	This study
R472 3detect	gccaataatcgttgtggacg	This study
LH56 5detect	cccgtccaataagggaagac	This study
LH56 3detect	caaccccatccccatgatgc	This study
HDA1 3DR	caatcttcggaagaggagtagtcttcaattgaatctaatatgaaatctactccttcatcgtggaattgtgagcggata	This study
HDA1 5DR	atgtcgactggtcaagaagaacatctagattctaagctagaaaatcaaatctcagaggatttcccagtcacgacgtt	This study
HDA1 5-detect	atagaagctaccattttcac	This study
HDA1 3-detect	agagatttcctagttatgtg	This study

The *HOS2* and *HDA1* complementation vectors pDDB503 and pDDB504 were constructed as follows. Wild-type *HOS2* and *HDA1* open reading frames (ORF), together with ∼1kb upstream and 0.5kb downstream of the *HOS2* and *HDA1* ORF, were amplified in high fidelity PCRs (Pfu Turbo DNA polymerase, Stratagene) from BWP17 DNA using primers HOS2 DDB78 comp 5′ and HOS2 DDB78 comp 3′, and HDA1 5′ comp, HDA1 5-1 comp, HDA1 3-1 comp and HDA1 3′ comp (Table 4). The resulting PCR products were *in vivo* recombined in *S. cerevisiae* strain L40 into a NotI/EcoRI-digested pDDB78 to generate plasmids pDDB503 and pDDB504.

### Media and growth conditions

*C. albicans* was routinely grown at 30°C in YPD (2% Bacto-peptone, 2% dextrose, 1% yeast extract). For selection of Ura^+^, Arg^+^, His^+^ or Trp^+^ transformants, synthetic medium without uridine, arginine, histidine or tryptophan was used (0.17% yeast nitrogen base without ammonium sulfate (Q-BioGene), 0.5% ammonium sulfate, 2% dextrose, and supplemented with a dropout mix containing amino and nucleic acids except those necessary for the selection [57]). M199 medium (Gibco BRL) was buffered at the indicated pH using 150mM HEPES. The filamentation assays in solid media were performed in M199 medium buffered at pH 8, SLAD (0.17% yeast nitrogen base without ammonium sulfate (Q-BioGene), 50 µM ammonium sulfate, 2% dextrose), Spider medium (1% mannitol, 1% nutrient broth, 0.2% K_2_HPO_4_, pH 7.2 before autoclaving) [58], embedded agar (2% Bacto-peptone, 2% sucrose, 1% yeast extract) [25], and synthetic medium supplemented with 4% bovine calf serum (BCS). The filamentation assays in liquid media were performed in M199 pH 8, Spider, YP +0.5% N-acetyl glucosamine (GlcNAc), and YP + 10% fetal bovine serum (FBS) (Gibco). Filamentation assays were conducted at 37°C except for embedded agar which was incubated at 23°C. The liquid assays for filamentation were performed as follows. Strains were grown overnight in liquid YPD at 30°C, pelleted, resuspended in an equal volume of PBS and diluted 1∶100 in M199 pH 8, Spider, YP+GlcNAc or YP+FBS. Samples were incubated at 37°C. The samples from Spider medium were gently sonicated to disrupt clumping. The percentage of cells forming germ tubes in M199 pH 8 medium at 60 min or in Spider medium at 45 min was determined by counting 300 cells/sample, in triplicate.

All media except that for selection of Ura^+^ transformants were supplemented with 80 µg/ml uridine. For solid media, 2% Bacto-agar was added, except for Spider medium and embedded agar which required 1.35% and 1% Bacto-agar, respectively.

### Microscopy

Pictures of colonies were taken using a Canon Powershot A560 digital camera on a Zeiss Opton microscope. Images of liquid cultures were captured using a Zeiss Axio camera, Axiovision 4.6.3 software (Zeiss), and a Zeiss AxioImager fluorescence microscope. All images were processed with Adobe Photoshop 7.0 software.
